# Editorial: AI with insight: explainable approaches to mental health screening and diagnostic tools in healthcare

**DOI:** 10.3389/fmed.2026.1798999

**Published:** 2026-02-13

**Authors:** Urška Smrke, Riku Klén, Izidor Mlakar, Satja Mulej Bratec, Inbar Levkovich

**Affiliations:** 1 Faculty of Electrical Engineering and Computer Science, University of Maribor, Maribor, Slovenia; 2 Turku PET Centre, University of Turku and Turku University Hospital, Turku, Finland; 3 Department of Psychology, Faculty of Arts, University of Maribor, Maribor, Slovenia; 4 Faculty of Medicine, University of Maribor, Maribor, Slovenia; 5 Faculty of Education, Tel Hai College, Upper Galilee, Israel

**Keywords:** artificial intelligence, depression, diagnosing, explainable artificial intelligence, mental health, monitoring, screening

Mental health conditions are often underrecognized and overlooked in healthcare, especially in primary healthcare (1). Primary care physicians frequently encounter patients presenting with mental health issues, either as standalone conditions or comorbidities to physical illnesses, placing them in a pivotal role for early detection and management. Screening for psychological disorders has been recognized as of great importance (2), however, several barriers to its implementation persist, including lack of staff, time constraints, accessibility restrictions and availability of services (3). Additionally, quality of self-reporting of internal states can be diminished due to reconstruction of memories, inattentive responding, social desirability, and reliance on cognitive heuristics (4–7).

With advancements in big data, artificial intelligence (AI) and technology, exciting new possibilities are arising in the screening for and supporting diagnosing of mental health conditions (8–11). In primary care, explainable artificial intelligence (XAI) offers solutions that can streamline mental health evaluations by providing accessible and interpretable tools that align with physicians' workflows (12, 13). Such approaches have the potential to overcome certain barriers to identifying persons with mental health disorders, as they can be cost-effective, efficient, and can provide an unintrusive assessment that can also support regular monitoring of persons at risk (14–16).

Especially promising are explainable, evidence-based approaches that translate existing knowledge into technology-supported screening and diagnosis of mental health conditions (12, 16). XAI tools can empower primary care providers by demystifying complex algorithms and enabling them to make informed decisions based on AI-generated insights (17). Additionally, these tools can facilitate interdisciplinary collaboration between primary care physicians and mental health specialists, bridging the gap in holistic care (10, 14).

This Research Topic brings together 11 multidisciplinary contributions that explore XAI in the realm of mental health and healthcare. Next, we briefly present each article and highlight how they together move the field from a “black-box” promise toward theoretically and clinically grounded, interpretable tools.

Several contributions in this Research Topic demonstrate how Generative AI (GenAI) and XAI can augment suicide prevention and AI-mediated support. Two studies evaluate a GenAI driven Question, Persuade, and Refer (QPR) suicide prevention simulation training aimed at mental health gatekeepers. Haber et al. provide support for its reliability, unbiasedness and ability to provide nuanced in-depth feedback to those in training, validating this approach as a valuable tool for advancing complex crisis intervention skills. In another study, Levkovich et al. evaluate the same simulation training from the perspective of trainees, finding large gains in their self-efficacy after training and generally favorable attitudes toward it. Within the suicide prevention domain, Grimland et al. focus on identifying suicide risk patterns via explainable Natural Language Processing (NLP) model. The study revealed several theoretical insights and highlighted the potential of AI-driven tools to support crisis counselors in real-time triage.

A second cluster focuses on interpretable models for individual-level screening of mental health disorders. Mekulu et al. presented a transparent four-feature speech model for depression screening, trained on brief conversational segments. Their model, optimized for deployment in resource-constrained settings, showed moderate discriminative performance and high sensitivity, and the article itself provides many insights on how semantic content can be used in screening, providing interpretable and clinically relevant information. Nozaki et al. extended explainable behavioral screening into the cognitive domain by applying machine learning to the self-administered rapid task to identify individuals presenting cognitive decline. Their approach suggests a non-invasive, resource efficient strategy in the field. In the cognitive domain, two articles focused on screening and classifying Alzheimer's disease, both harnessing imaging data collection techniques. Reddy et al. presented self-attention-based vision used to predict Alzheimer's disease via magnetic resonance imaging (MRI) images with promising results, presenting an important step toward robust and clinically applicable models in this area. Slimi et al. present a hybrid convolutional neural network-spiking neural network aimed at classifying Alzheimer's disease stages. This study suggests possibilities to improve early detection of Alzheimer's disease in a computationally efficient and biologically relevant approach.

The third topic explored AI approaches beyond detection and classification and provides a critical perspective on the MIT-OpenAI RCT study that explored AI-supported therapy. Ophir et al. provide a broad perspective on the evaluation of early evidence in this domain and advise on several points in the rapidly developing area of AI-supported therapy.

Finally, three articles provide conceptual and methodological scaffolding for XAI in explainable mental health. Taskynbayeva and Gutoreva present a systematic review of anxiety-prediction machine learning (ML) models synthesizing the evidence from 19 studies. Findings offer support to the effectiveness of ML for early anxiety detection. Next, Močnik et al. present a review of 24 review articles that focused on multimodal cues of mood, anxiety or borderline personality disorders, and provides a valuable synthesis of observable speech, language, facial, physiological, and digital-behavioral markers of these conditions, positioning them as promising for exploitation in developing XAI algorithms for early detection and monitoring of said conditions. Finally, Yang et al. propose a novel XAI framework for economic mental-health time-series forecasting. This promising framework illustrates how XAI can support not only individual-level screening but also policy-relevant monitoring of mental health trends.

**Figure 1:**
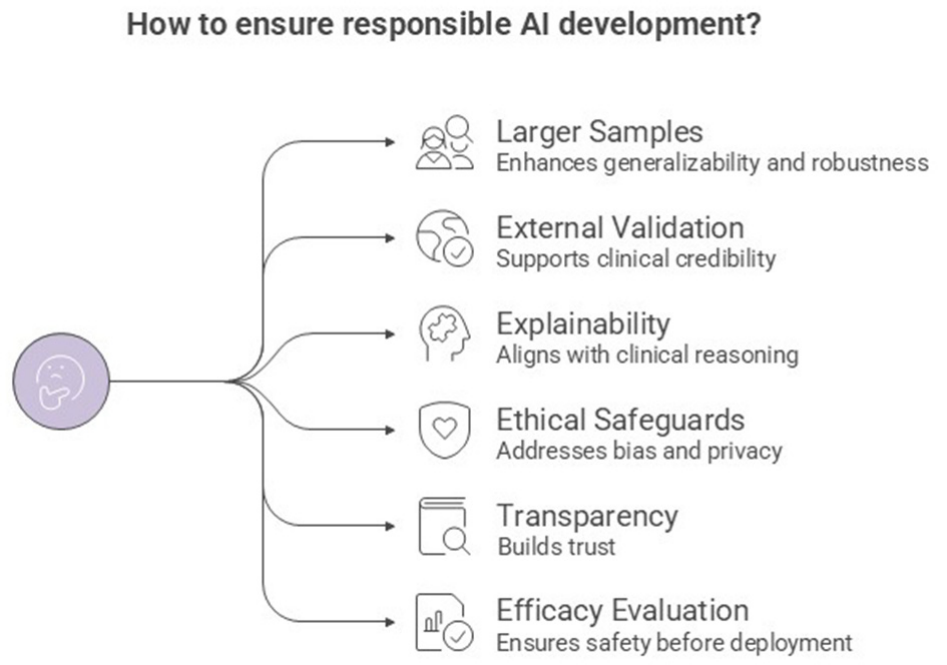
Recommendations for responsible AI development.

Across the contributions to this Research Topic, several priorities emerge for advancing XAI approaches in mental health care (Figure 1). There is a strong consensus on the need to move beyond small and homogeneous samples toward larger, richer datasets to improve the generalizability and robustness of the models. Future research should prioritize external validation, and tightly align explainability with clinical reasoning, to transfer high accuracy rates into clinically trustworthy deployment, while not forgetting crucial ethical aspects, such as bias, cultural blind spots and erosion of human connections.

The articles demonstrate critical progress in embedding transparency, interpretability, and clinical grounding within AI systems for screening and diagnosing mental health. Across suicide prevention, depression and cognitive screening, dementia imaging, anxiety prediction, and population-level forecasting, they demonstrate that model transparency and performance can coexist. As such, the XAI approaches have a promising potential to build on these foundations and substantially support healthcare services to detect problems earlier, personalize support, and substantially improve access and quality of care.
